# Effect of Dietary Protein Levels on Performance and Health Status of Adult Companion Rabbits

**DOI:** 10.3390/ani15192784

**Published:** 2025-09-24

**Authors:** Bianca Palumbo, Antonella Dalle Zotte

**Affiliations:** Department of Animal Medicine, Production and Health, University of Padova, Agripolis, Viale dell’Università 16, 35020 Legnaro, Padova, Italy; biancafederica.palumbo@phd.unipd.it

**Keywords:** sex-specific nutrition, energy balance, metabolic demand, digestibility, fat deposition, coloured dwarf rabbit breed

## Abstract

Three isoenergy diets with different crude protein (CP) levels (H = 175, M = 173, and L = 165 g/kg, as is), were fed to 117 sex balanced rabbits (coloured dwarf breed) from 28 to 45 weeks of age. Feed intake, body weight, and fatness were unaffected by the dietary treatment. By reducing dietary CP content, rabbits in group L had a significantly reduced proportion of digestible protein (DP) relative to maintenance requirements compared to group H; *p* < 0.001), but still exceeding their DP needs. The initial body weight of females was significantly greater than that of males, a difference maintained throughout the study period. Males consumed more feed than females (86.0 vs. 75.2 g/d, *p* < 0.001) and at 45th week, the skin fold width of males was higher than that of females (*p* < 0.001). DP balance remained positive in both sexes but was higher in males than females (+3.37 vs. +2.50 g DP/d; *p* < 0.001). Contrarily, the energy balance was positive in males (+57.9 kJ DE/d) and negative in females (−20.3 kJ DE/d). In conclusion, a dietary CP level of 165 g/kg is considered adequate for adult companion rabbits at maintenance. It is therefore advisable to diversify diets by sex to better meet their nutritional requirements.

## Simple Summary

Feeding adult companion rabbits with diets supplying adequate protein levels may help maintain good health without overloading them with nutrients they do not need. In this study, 117 adult companion rabbits were fed balanced diets, differing only for their crude protein content. Results showed that the lowest protein diet tested was enough to support health, body maintenance, and energy and nutrient digestibility, while the higher protein diets caused an excess of digestible protein relative to available energy. Results highlight that male and female rabbits have different nutritional needs at adult stage. Feeding both sexes the same diet may lead to energy and nutrients imbalances in one or both sexes. Tailoring diets based on sex can improve feed efficiency, body condition, and overall well-being in companion rabbits. These findings offer practical advice for pet owners, veterinarians, and feed producers to improve rabbit care through better, more targeted feeding strategies.

## 1. Introduction

Rabbits are bred for several purposes such as source of meat, laboratory experiments, wool and fur production and in recent years they have become increasingly popular as pet and companion animals [1,2]. Husbandry and nutrition are key factors for maintaining healthy rabbits. Although extensive studies have been conducted on farmed and laboratory rabbits, these findings cannot be directly applied to companion ones [3,4]. Pet rabbits, indeed, have a longer life expectancy of about 5–12 years. During this time, it is important to pay attention to their diet, living environment, and overall care provided by the owner [5]. Many common conditions in companion rabbits, such as gastrointestinal issues, dental disease, and obesity, are often directly caused by a prolonged inadequate nutrient supply [6]. Pet rabbits, like those reared for production, have nutritional requirements that vary according to their physiological state, and diet plays a key role in meeting these needs. Optimal crude fibre levels in rabbits diet range from 20% to 32%, with a minimum of 12% required to maintain proper digestive function [7,8,9,10]. Fat is typically included in the diet at optimal levels of 2.5% to 4%, added to pellets to provide energy, improve palatability, and reduce dustiness [8,9]. Regarding crude protein (CP), its levels in rabbit’s diet significantly influences growth performance, carcass yield, and organ weights. A CP content between 15% and 16% is generally considered appropriate for optimal growth, while for lactating does, a level of about 18% is recommended [11,12]. In the study of [13] increasing the CP from 14% to 17% linearly increased the growth rate of the rabbits along with the final BW, while the feed conversion ratio (FCR) decreased. Similarly, [14] reported that raising the CP level up to 20% increased the average daily gain (ADG) and reduced the FCR.

In addition to conventional feed components, growing interest has been shown in the use of functional foods in rabbit diets to further improve health and prevent disease. For instance, the inclusion of natural products such as Spirulina (*Arthrospira platensis*) and Thyme (*Thymus vulgaris* L.) has attracted attention due to their potential health benefits and positive medical properties [15]. Spirulina, a blue-green algae, is considered a valuable natural health food rich in easily digestible protein, about 55–65% and essential amino acids [16,17]. It is also a source of essential fatty acids such as γ-linolenic acid (GLA, C18:3 n-6) [18], chlorophyll, carotenoids, minerals, carbohydrates, sterols, and pigments that contribute to its antioxidant properties [19]. Similarly, thyme and thyme essential oil are well known for their antioxidant and antimicrobial properties [20].

Rabbits generally have a high metabolic rate, with an energy requirement for maintenance of about 430 kJ ED/d/kg metabolic live weight (LW^0.75^), and further specific requirements for growing rabbits and reproducing does [8,9]. Under good sanitary conditions, growing rabbits consume enough feed to meet their energy requirements for both maintenance and growth. Their voluntary energy intake is proportional to their metabolic live weight (LW^0.75^), averaging 950 kJ of digestible energy (DE) per day per kg LW^0.75^, approximately 2.2 times their maintenance energy requirement. On the other hand, reproducing does have additional energy requirements for pregnancy and/or lactation, which are often not fully met through voluntary feed intake [12]. Sexual dimorphism influences both feed intake and energy utilization, primarily due to differences in nutrient partitioning and physiological requirements. In growing rabbits, DE intake is typically allocated 45% for maintenance and 55% for growth [9,12]. In adults, maintenance energy accounts for approximately 40–50% of total DE intake, though this proportion varies depending on sex, physiological status, and age [21]. For companion rabbits, dietary DE ranges between 9 and 10.5 MJ DE/kg. The energy-to-protein ratio can be estimated at approximately 98 kJ per gram of digestible protein (DP). Therefore, for diets containing 102–110 g DP/kg, an energy level of 10–10.5 MJ DE/kg is considered appropriate [22]. Specifically, the DP to DE ratio should range from 10.5 to 11.5 g MJ^−1^ for young rabbit and bucks and from 11.5 to 12.5 g MJ^−1^ for reproducing does [23,24]. However, the proximate composition of commercial pet rabbit diets available on the Italian market has been shown to be highly heterogeneous and often does not meet the nutritional requirements of pet rabbits, particularly with respect to protein and energy levels [25]. In some cases, these diets are not suitable for the physiological needs of the animals.

Given these considerations, the aim of the present research was to evaluate the effects of diets with different level of CP on live performance, apparent digestibility of energy and nutrients, and on the health status of adult companion rabbits. The experiment was conducted on both males and females to assess potential sex-related differences in their physiological and nutritional responses.

## 2. Materials and Methods

### 2.1. Rearing Building, Equipment and Animals

The study was carried out at the experimental rabbitry of the University of Padova (Italy) using a building equipped with cages for reproduction does (400 × 600 × 350 mm) and for fattening rabbits (240 × 400 × 280 mm). The cages were made of galvanized wire net equipped with automatic drinkers and manual feeders.

One hundred and seventeen coloured dwarf rabbits aged 28 weeks with an average weight of 1773 ± 7.2 g were randomly assigned to three experimental groups of 39 animals each (L: 21 males and 18 females; M: 20 males and 19 females; H: 21 males and 18 females) homogenised by live weight (LW) and standard deviation. The animals were housed individually under standard condition at temperature of 19 ± 2 °C.

### 2.2. Experimental Diets and Periods

Three experimental diets were formulated with increasing levels of crude protein (CP): a low CP diet (L) containing 165 g/kg feed, a medium CP diet (M) containing 173 g/kg feed, and a high CP diet (H) containing 175 g/kg feed.

The experimental diets were made by substituting fibrous raw materials partially with sugar beet pulp (SBP). The ingredients of the diet, in decreasing order, were: polyphyte grass hay, dehydrated alfalfa (15% CP), sunflower meal, sugar beet pulp, wheat straw, wheat bran, molasses. The diets were enriched with spirulina (*Arthrospira platensis*) in dose to satisfy the protein requirement (0.98–2.49%), thyme leaves (*Thymus vulgaris*) 2%, extruded flax 2–4%, and yucca (*Yucca schidigera*) 0.0125% as a natural deodoriser. The chemical composition and the nutritional value of the three diets are showed in Table 1.

The experimental diets were pelleted and stored in paper bags. The average pellet dimensions were 12.66 × 3.64 mm. After an adaptation period to both the diets and the cages, the rabbits were fed ad libitum with the three experimental diets for 18 weeks. The animals had free access to clean drinking water and to individual feeders. Feed was weighed and distributed twice a week. The uneaten feed was weighed before each new distribution to determine feed consumption. Twice a week, the rabbits were weighed individually to monitor weight gain, feed intake, and feed efficiency, in order to assess performance. To evaluate the fattening status of the animals, skin fold thickness was measured at 28th and 45th week using a digital calliper (H 4215/3, 0–150 mm, ABC Tools, Milan, Italy).

### 2.3. Digestibility Trial

Twenty-four sex-mixed (14 females and 10 males) rabbits (8 rabbit per diet, balanced by sex) of 32 weeks of age were randomly selected to perform a digestibility trial. The digestibility trial was conducted according to the European standardised method [26]. Rabbits had an average body weight of 1835 ± 16 g. The faecal apparent digestibility of dry matter (DM), gross energy, CP, ether extract (EE), crude fibre (CF) and its fractions, neutral detergent fibre (NDF), acid detergent fibre (ADF), acid detergent lignin (ADL) and starch were determined. After a seven-day adaptation period to the feed and to the individual digestibility cages, DM intake and total faecal output were recorded for each rabbit over a four-day collection period. Faeces were collected using nets placed under the cages, equipped with a plate deflector to prevent urine contamination. The daily faeces were placed in labelled polyethylene bags and stored at −20 °C. At the end of the trial, all faecal samples were dried at 80 °C for 48 h and ground to 1 mm for subsequent analysis.

### 2.4. Chemical Analysis

The [27] procedures were used to determine the following parameters of experimental diets and faeces: DM (method no. 934.01), ash (method no. 924.05), CP (method no. 976.05), CF (CF, 978.10), and its fractions: NDF (NDF, 2002.04), ADF and ADL (method no. 973.18) by using the Ankom Technology (Macedon, NY 14502) with filter bags (F57). Ether extract (method no. 920.39) was determined after acid- hydrolysis, whereas starch with amyloglucosidase α-amylase method (method no. 996.11). Gross energy (GE) was determined by adiabatic bomb calorimetry [28]. DE was calculated based on the results of the digestibility trial. Mineral analysis (Ca, K, Na, P) was performed by inductively coupled plasma optical emission spectrometry (ICP-OES; Spectro Ciros Vision EOP) after microwave digestion (method no. 999.10), whereas chlorine was determined according to the AOAC procedure method no. 943.01. Vitamin E content was analysed following the methods described in the [29]. Fatty acid (FA) profile of experimental diets was analysed by gas chromatography (GC), after extraction with an accelerated solvent extraction (ASE) system using petroleum ether as the solvent, according to the method described by [30]. GC was performed using an automated system (Shimadzu GC 17A, Kyoto, Japan) equipped with a flame ionisation detector and a Supelco Omegawax 250 capillary column (30 m × 0.25 mm ID). The operating conditions were as follows: injection temperature, 260 °C; helium (He 6.0) flow rate, 1.30 mL/min (linear velocity: 35 cm/s at 220 °C). Fatty acids were identified by comparing retention times with those of authentic FA methyl ester standards (Mix C4–C24, 18919-1AMP, Supelco, Bellefonte, PA, USA). Results were expressed as a percentage (*w*/*w*) of total fatty acid methyl esters. All analyses were performed in duplicate.

### 2.5. Statistical Analysis

All variables were initially tested for normality using the PROC UNIVARIATE procedure in SAS software 9.1.3 [31]. Normal distribution was assessed using the Shapiro–Wilk test, and homogeneity of variances was evaluated with Levene’s test. Since the assumptions of normality and homoscedasticity were satisfied for all data related to growth performance, feed intake, diet digestibility, and skinfold width, analysis of variance (ANOVA) was conducted using the PROC GLM procedure in SAS. The model included diet (L, M, and H), sex and their interaction as fixed effects. Health status data (mortality %, morbidity %) were analysed using a chi-square test via the PROC GENMOD procedure, with diet included as a fixed effect. Differences among least squares means (LS-means) were assessed using the Bonferroni-adjusted *t*-test. Statistical significance was considered at *p* ≤ 0.05.

## 3. Results and Discussion

### 3.1. Digestibility Trial

Data from the digestibility trial are presented in Table 2. The experimental diets had no effect on the rabbits’ feed intake during the digestibility period. However, sex significantly influenced feed intake when expressed as g DM per kg of LW, with males consuming more than females (0.15 vs. 0.13 g DM/kg LW; *p* < 0.05). Findings of the present study indicate that the three different diets administered to companion rabbits affected the apparent faecal digestibility of certain components. In the present study, no significant differences in CP digestibility were observed with variations in dietary CP content, which is consistent with the findings of previous studies [32,33]. In contrast, the higher EE content of the diet significantly affected the apparent faecal digestibility of EE, which was higher in rabbits fed the M and L diets compared to those on the H diet (80.8% vs. 76.7% on average, respectively; *p* < 0.01). This is in line with findings of other studies, where the addition of fat, especially vegetable oils, increased the apparent digestibility of EE [34,35,36]. A similar pattern was observed for CF digestibility (22.5% vs. 17.6% on average, respectively; *p* < 0.01) and for the ADF fraction (29.3% vs. 25.7% on average, respectively; *p* = 0.0002). Rabbit fed with L diet also showed higher digestibility of NDF (36.8% vs. 31.9%, on average; *p* < 0.0001) cellulose (37.3% vs. 32.9%, on average; *p* < 0.0001) and hemicellulose (45.6% vs. 39.8%, on average; *p* = 0.0001), compared to those fed the M and H diet. Digestibility of fibre in rabbits is closely related to the type of dietary fibre and the proportion of fine particles (<0.315 mm) [10]. In the present experiment, CF content was similar across all three diets (174 g/kg). However, diets M and H had higher levels of ADL. The increased lignin content in diet H may have reduced crude fibre digestibility in rabbits fed this diet, as lignin is an indigestible component of fibre [37]. Sex significantly influenced the NNCC digestibility, with females showing higher values than males (*p* < 0.05). A limitation of the present study is the lack of consideration of a broader health-related parameters such as blood metabolites, metabolic biomarkers, or faecal microbiota composition. Future studies incorporating a more comprehensive set of physiological and metabolic measurements would provide a more robust assessment of the dietary intervention and its implications for overall animal health.

### 3.2. Live Performance and Health Status

**Figure 1:**
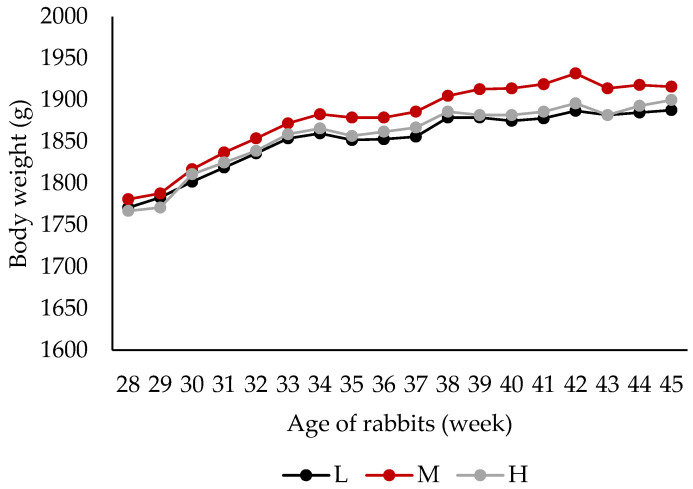
Evolution of body weight (g) of companion rabbits during the trial according to diets. In all time points, diet effect: *p* > 0.10. L = low crude protein content, M = medium crude protein content, H = high crude protein content.

**Figure 2:**
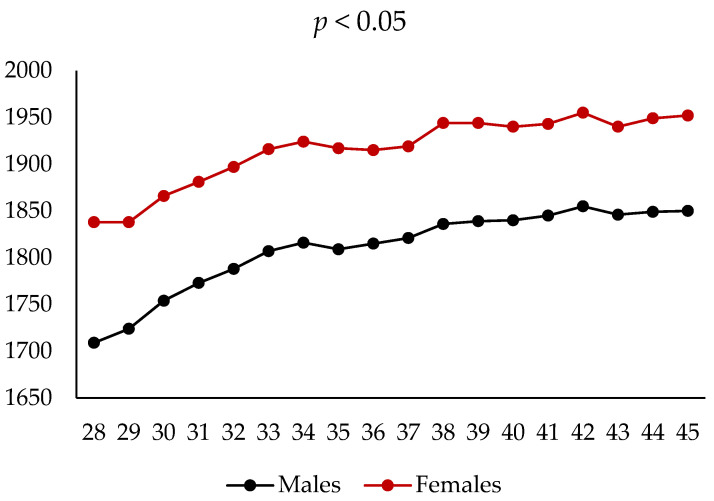
Evolution of body weight (g) of companion rabbits during the trial according to sex. In all time points, diet effect: *p* < 0.05.

Figure 1 and Figure 2 show the evolution of LW in rabbits throughout the trial, according to diet and sex. No significant effect of the experimental diets was observed on growth, with similar BW recorded across the three treatment groups. In contrast, sex had a marked effect on LW, with females being heavier than males since the start of the trial (1838 g vs. 1709 g; *p* < 0.01). This difference persisted until the end of the study at 45 weeks of age (1952 g vs. 1850 g; *p* < 0.05).

Weekly feed intake, evaluated throughout the trial (Table 3), was significantly affected by the experimental diets. Specifically, rabbits fed the H diet showed higher intake from week 35 to week 42 and again from week 44 to week 45. In contrast, the lowest values were recorded in rabbits fed the L diet, while those on the M diet generally showed intermediate intake levels (84.4 g/week vs. 73.4 g/week on average, respectively; *p* < 0.0001). However, no significant differences in overall feed intake were observed among the dietary groups over the entire period from week 28 to week 45. Sex had a significant effect on feed intake during specific periods: the first week of rearing, from week 40 to week 43, and the final week of rearing. During these times, males showed higher feed intake than females (82.6 g/week vs. 74.2 g/week on average, respectively; *p* < 0.001). Consequently, total feed intake from week 28 to week 45 was also significantly higher in males compared to females (86.0 g/week vs. 75.2 g/week, respectively; *p* < 0.05). Although male rabbits had lower LW compared to females, their higher feed intake during specific rearing periods may be attributed to differences in appetite regulation according to physiological status (maintenance, growth, reproduction). Adult males appear to regulate feed intake similarly to rabbit does, reaching satiety only after a greater intake, which favours increased fat deposition. Furthermore, testosterone, the predominant male sex hormone, has been shown to increase metabolic rate and stimulate feed intake in various species [38].

Unlike feed intake, body weight gain in rabbits was not significantly affected by either the experimental diets or sex (Table 4). A significant difference was observed only during the second week of breeding, when rabbits fed the H diet showed a higher weight gain compared to those in the L group (5.76 g/day vs. 2.73 g/day, respectively; *p* < 0.05). A difference was also noted in weeks 42–43 likely due to temperature fluctuations that may have affected weight gain during that period.

Animal fattening, assessed through changes in skin fold width, is presented in Table 5. Skin fold thickness measured at weeks 28 and 45 was not affected by the experimental diets. However, sex significantly influenced fattening at both time points. Specifically, males had significantly greater skin fold thickness than females at both week 28 (3.49 mm vs. 3.08 mm; *p* < 0.001) and week 45 (4.35 mm vs. 3.56 mm; *p* < 0.001). Consequently, the increase in skin fold width over the trial period was also significantly greater in males compared to females (0.85 mm vs. 0.48 mm; *p* < 0.05). This finding indicate that male rabbits accumulated significantly more subcutaneous fat than females. In New Zealand White rabbits, males have been observed to reach sexual maturity later than females, which may explain differences in growth patterns and greater lipid deposition, findings that support the results of the present study [39]. Moreover, sex-based differences in adipose tissue distribution and gene expression, modulated by variations in estrogen receptor ratios, have been demonstrated in both rodent and non-human primate models, suggesting the existence of conserved endocrine pathways that influence fat storage patterns across species [40].

No mortality was recorded throughout the trial. Additionally, no differences were observed among the groups in terms of morbidity (Table 6).

### 3.3. Protein and Energy Diet Balance

Table 7 shows the DP and DE balance evaluated in the experimental groups of rabbits during the final two weeks of the trial. Weeks 44 and 45 were selected for this analysis, as growth during this period was either negligible or absent (see Table 4). Females showed a significantly higher final and metabolic LW compared to males (*p* = 0.03), which resulted in higher daily maintenance energy requirement (*p* = 0.03). Feed intake was influenced by dietary protein level (*p* = 0.04), being highest in rabbits fed the H diet, while no differences were observed between sexes.

DE intake did not differ among groups; however, DE balance was significantly affected by sex: males showed a positive DE balance while females a negative one (+57.9 vs. −20.3 kJ/d, respectively; *p* = 0.0006). In contrast, DP intake was affected by diet, with the highest values in rabbits fed the H diet and the lowest in those fed the L diet (10.0 vs. 8.36 g/d; *p* = 0.0005). DP balance was positive across all groups and significantly affected by both diet and sex. It was greatest in rabbits fed the H diet, intermediate in those fed the M diet, and lowest in the L group (*p* < 0.0001). Moreover, males exhibited a significantly higher DP balance than females (*p* = 0.003), although it remained positive in both sexes. These findings suggest that the diets had a disproportionately high DP to DE ratio, indicating an excess of digestible protein relative to available energy. This imbalance likely led to insufficient energy supply in females, resulting in a negative DE balance. Only in the L diet, where energy and protein were more proportionally balanced, did females achieve a positive energy balance.

This highlights that male and female adult companion rabbits have different energy and protein requirements, which should be considered in diet formulation. Notably, this sex-based divergence in nutritional needs becomes more pronounced after 15 weeks of age [41]. The observed differences in adult energy requirements between sexes may be attributed to hormonal influences, variations in body composition, or behavioural factors. Additionally, females mature earlier than males and reach their maximum growth rate sooner [39]. Therefore, implementing sex-specific feeding strategies may improve growth uniformity and overall production efficiency.

## 4. Conclusions

The present study provided valuable insights for optimising feed formulation in adult coloured dwarf companion rabbits. Results indicated that a dietary protein level of 165 g/kg (as fed) is adequate, since higher protein levels were associated with an unbalanced DP to DE ratio. This imbalance led to insufficient available energy and a negative DE balance in females, despite a positive DP balance across all groups. Furthermore, findings revealed significant sex-based differences in live performance, body fatness, and energy balance, emphasising the importance of sex-specific nutritional strategies. Females were consistently heavier than males, but males displayed higher FI during adult life, reflecting increased metabolic and behavioural demands. In addition, males accumulated significantly more subcutaneous fat than females, regardless of diet, indicating that males have a different body weight gain composition, more oriented towards lipid deposition. Overall, these findings emphasise the importance of considering sex-specific nutritional requirements in adult companion rabbits. Feeding strategies that account for both dietary composition and sex-related differences may control obesity, and enhance overall health and welfare in adult companion rabbits.

## Figures and Tables

**Table 1:** Chemical composition and nutritive value of experimental diets (as fed).

	Diets ^1^		
	L	M	H
Dry matter, g/kg	882	887	886
Crude protein, g/kg	165	173	175
Ether extract, g/kg	40.0	44.0	32.0
Ash, g/kg	77.0	87.0	88.0
Starch, g/kg	69.0	73.0	73.0
Crude fibre, g/kg	173	176	173
Neutral detergent fiber g/kg	352	353	342
Acid detergent fiber, g/kg	205	214	208
Acid detergent lignin, g/kg	34.0	38.0	37.0
Gross Energy, MJ/kg	16.5	16.5	16.2
Ca, g/kg	9.70	12.1	11.0
P, g/kg	4.70	5.00	4.70
K, g/kg	15.0	16.2	15.7
Na, g/kg	1.80	2.20	3.70
Ca:P	2.06	2.42	2.34
Vitamin D_2_, UI/kg	1326	1310	1108
Vitamin D_3_, UI/kg	4594	8363	4006
Lysine, mg/100 g	1.05	1.10	1.83
Tryptophane, mg/100 g	0.27	0.27	0.29
Nutritive value:			
Digestible Energy (DE), MJ/kg	10.0	9.70	9.60
Digestible Protein (DP), g/kg	120	124	128
DP to DE ratio, g/MJ	12.0	12.7	13.4

^1^ L = low crude protein content, M = medium crude protein content, H = high crude protein content.

**Table 2:** Feed intake, faecal dry matter, and apparent faecal digestibility in companion rabbits fed the experimental diets.

	Diet ^1^ (D)			Sex (S)			*p* Value ^2^	RSD ^3^
	L	M	H	Male	Female	D	S	
No. of rabbits	8	8	8	10	14			
Dry matter excreta, g	90.0	111	110	110	97.8	ns	ns	24.0
Feed intake, g	252	295	300	296	269	ns	ns	56.0
Feed intake, g/DM	222	260	265	261	237	ns	ns	49.0
Feed intake, g DM/kg LW	122	141	145	146	126	ns	<0.05	0.20
Apparent faecal digestibility:								
Dry matter, %	59.5	57.6	58.6	58.2	58.9	ns	ns	1.70
Organic matter, %	59.7	57.5	58.2	58.2	58.8	ns	ns	1.70
Crude protein, %	72.7	71.4	73.4	72.8	72.2	ns	ns	2.90
Ether extract, %	81.4 ^A^	80.2 ^A^	76.7 ^B^	79.0	79.9	<0.01	ns	2.20
Starch, %	98.1	97.7	97.7	97.7	97.9	ns	ns	0.40
Crude Fibre (Weende), %	22.5 ^A^	22.4 ^A^	17.6 ^B^	20.9	20.7	<0.01	ns	4.00
Neutral-detergent fibre (NDF), %	36.8 ^A^	32.9 ^B^	30.9 ^B^	33.8	33.3	<0.0001	ns	2.80
Acid-detergent fibre (ADF), %	30.5 ^A^	28.0 ^A^	25.7 ^B^	28.2	27.9	0.0002	ns	1.70
Cellulose (ADF-ADL), %	37.3 ^A^	33.6 ^B^	32.2 ^B^	34.2	34.6	<0.0001	ns	1.90
Hemicelluloses (NDF-ADF), %	45.6 ^A^	40.6 ^B^	38.9 ^B^	42.0	41.4	0.0001	ns	1.80
NNCC, % ^4^	80.2	80.1	82.2	79.5	82.2	ns	<0.05	2.30
Gross energy, %	60.7	58.8	59.0	59.3	59.8	ns	ns	1.80
Ca, %	52.9	53.4	52.9	51.9	54.2	ns	ns	3.70
P, %	12.1	11.5	12.2	12.5	11.4	ns	ns	5.30

^1^ L = low crude protein content, M = medium crude protein content, H = high crude protein content; ^2^ ^A, B^: *p* < 0.01; ns: not significant; ^3^ Residual Standard Deviation; ^4^ NNCC: Non-Nitrogenous Cellular Content = Organic matter–NDF–crude protein.

**Table 3:** Feed intake (g/d) of companion rabbits during the trial (from 28 to 45 weeks of age).

	Diet ^1^ (D)			Sex (S)		*p* Value ^2^		RSD ^3^
	L	M	H	Male	Female	D	S	
No. of rabbits	39	39	39	60	57			
Weeks of age:								
28–29	83.7	86.3	82.9	91.6	77.0	ns	<0.001	20.5
29–30	87.4	95.0	100	95.3	93.1	ns	ns	23.6
30–31	86.9	94.1	92.6	94.0	88.4	ns	ns	20.1
31–32	86.3	91.4	92.7	91.8	88.5	ns	ns	18.9
32–33	87.3	91.8	94.7	93.2	89.3	ns	ns	18.0
33–34	83.9	91.2	94.3	91.9	87.7	ns	ns	16.1
34–35	81.3	87.9	89.4	87.3	85.1	ns	ns	15.8
35–36	74.4 ^B^	81.0 ^AB^	87.7 ^A^	82.7	79.5	<0.01	ns	17.7
36–37	75.4 ^b^	83.3 ^ab^	86.1 ^a^	84.6	78.6	<0.05	ns	18.4
37–38	78.5 ^b^	85.7 ^ab^	88.9 ^a^	86.3	82.4	<0.05	ns	17.8
38–39	77.6	86.2	84.5	85.6	79.8	ns	ns	16.9
39–40	70.0 ^B^	79.8 ^B^	80.4 ^A^	78.2	74.3	<0.01	ns	15.8
40–41	71.4 ^B^	81.2 ^A^	83.4 ^A^	82.5	74.8	<0.001	<0.01	14.4
41–42	77.6 ^b^	82.7 ^ab^	86.1 ^a^	85.6	78.7	<0.05	<0.05	14.4
42–43	72.8	75.3	77.0	78.1	71.9	ns	<0.05	14.7
43–44	72.4	74.8	77.9	76.9	73.2	ns	ns	16.1
44–45	66.8 ^B^	72.0 ^AB^	78.0 ^A^	75.4	69.2	<0.01	<0.05	15.1
28–45	77.2	82.9	82.1	86.0	75.2	ns	<0.05	14.4

^1^ L = low crude protein content, M = medium crude protein content, H = high crude protein content; ^2^ ^A, B^: *p* < 0.01; ^a, b^: *p* < 0.05; ns: not significant; ^3^ Residual Standard Deviation.

**Table 4:** Weight gain (g/d) of companion rabbits during the trial (from 28 to 45 weeks of age).

	Diet ^1^ (D)			Sex (S)		*p* Value ^2^		RSD ^3^
	L	M	H	Male	Female	D	S	
No. of rabbits	39	39	39	60	57			
Weeks of age:								
28–29	1.82	1.01	0.52	2.19	0.04	ns	ns	6.19
29–30	2.73 ^b^	4.06 ^ab^	5.76 ^a^	4.32	4.05	<0.05	ns	4.83
30–31	2.30	2.85	2.01	2.69	2.08	ns	ns	3.73
31–32	2.45	2.46	1.84	2.21	2.28	ns	ns	3.85
32–33	2.57	2.54	2.93	2.62	2.74	ns	ns	3.24
33–34	0.92	1.69	0.97	1.28	1.11	ns	ns	3.15
34–35	−1.18	−0.57	−1.18	−1.03	−0.92	ns	ns	3.34
35–36	0.17	−0.01	0.67	0.93	−0.38	ns	ns	4.49
36–37	0.46	0.97	0.76	0.84	0.62	ns	ns	4.18
37–38	3.30	2.72	2.68	2.21	3.59	ns	ns	3.84
38–39	−0.01	1.04	−0.53	0.31	0.03	ns	ns	3.77
39–40	−0.68	0.28	−0.10	0.22	−0.55	ns	ns	3.80
40–41	0.49	0.65	0.62	0.77	0.41	ns	ns	3.71
41–42	1.33	1.92	1.35	1.41	1.66	ns	ns	3.85
42–43	−0.68 ^b^	−2.58 ^a^	−1.97 ^ab^	−1.37	−2.12	<0.05	ns	3.31
43–44	0.41	0.53	1.61	0.49	1.21	ns	ns	3.86
44–45	0.40	−0.26	1.04	0.18	0.60	ns	ns	3.36
28–45	0.99	1.13	1.12	1.19	0.97	ns	ns	1.02

^1^ L = low crude protein content, M = medium crude protein content, H = high crude protein content; ^2^ ^a, b^: *p* < 0.05; ns: not significant; ^3^ Residual Standard Deviation.

**Table 5:** Skin fold width, measured at the 28th and the 45th week of age.

	Diet ^1^ (D)			Sex (S)		*p* Value		RSD ^2^
	L	M	H	Male	Female	D	S	
No. of rabbits	39	39	39	60	57			
Skin fold width at 28th week, mm	3.24	3.36	3.25	3.49	3.08	ns	<0.001	0.49
Skin fold width at 45th week, mm	3.85	3.92	4.08	4.35	3.56	ns	<0.001	0.63
Skin fold width change, mm	0.61	0.56	0.83	0.85	0.48	ns	<0.05	0.77
Skin fold width change, %	20.9	19.2	27.6	26.9	18.3	ns	ns	25.1

^1^ L = low crude protein content, M = medium crude protein content, H = high crude protein content; ^2^ Residual Standard deviation.

**Table 6:** Mortality, morbidity and sanitary risk (%) of companion rabbit during the trial.

	Diet ^1^ (D)			*p* Value	RSD ^2^
	L	M	H		
No. of rabbits	39	39	39		
Mortality, %	0	0	0		
Morbidity, %	33.9	35.6	32.4	ns	5.81

^1^ L = low crude protein content, M = medium crude protein content, H = high crude protein content; ^2^ Residual Standard deviation.

**Table 7:** Protein and energy balance of male and female experimental groups refers to the last two weeks of rearing (44th–45th week of age).

	Diet ^1^ (D)			Sex (S)		*p* Value ^2^		RSD ^3^
	L	M	H	Male	Female	D	S	
No. of rabbits	39	39	39	60	57			
Final live weight (45th week)	1883	1916	1902	1846	1950	ns	0.03	0.26
Metabolic live weight (kg^0.75^)	1605	1626	1615	1581	1650	ns	0.03	0.17
Maintenance energy requirement, kJ DE/d	690	699	694	680	709	ns	0.03	71.7
Feed intake, g/d (44th to 45th week)	69.7 ^b^	71.5 ^ab^	77.9 ^a^	75.0	71.0	0.04	ns	14.6
DE intake, kJ/d	698	695	745	737	689	ns	ns	142
DE > maintenance energy, kJ/d	7.53	−3.69	52.5	57.9	−20.3	ns	0.0006	119
DP intake, g/d	8.36 ^C^	8.84 ^B^	10.0 ^A^	9.37	8.77	0.0005	ns	1.81
Maintenance DP requirement, g/d	6.10	6.18	6.14	6.00	6.27	ns	0.03	0.63
DP > maintenance DP, g/d	2.26 ^B^	2.66 ^AB^	3.86 ^A^	3.37	2.50	<0.0001	0.003	1.56

^1^ L = low crude protein content, M = medium crude protein content, H = high crude protein content; ^2^ ^A, B, C^: *p* < 0.01; ^a, b^: *p* < 0.05; ns: not significant; ^3^ Residual standard deviation.

## Data Availability

The data sets analysed in the present study are available from the corresponding author upon request.

## Abbreviations

The following abbreviations are used in this manuscript:

CP	Crude protein
FCR	Feed conversion ratios
LW	Live weight
LW^0.75^	Metabolic live weight
DE	Digestible energy
DP	Digestible protein
L	Low crude protein diet
M	Medium crude protein diet
H	High crude protein diet
SBP	Sugar beet pulp
DM	Dry matter
EE	Ether extract
CF	Crude fiber
NDF	Neutral detergent fiber
ADF	Acid detergent fiber
ADL	Acid detergent lignin
GE	Gross energy
ASE	Accelerated solvent extraction
GC	Gas chromatography
ANOVA	Analysis of variance
